# Primary Breast Burkitt's Lymphoma in an HIV-Infected Woman

**DOI:** 10.1155/2015/792041

**Published:** 2015-05-27

**Authors:** Bangaly Traoré, Marie-Eve Fondrevelle, Mamoudou Condé, Catherine Chassagne-Clément, Tidiane Kourouma, Ahmed Monzomba Keita, Moussa Koulibaly, Bakary Sidiki Sylla

**Affiliations:** ^1^Surgical Oncology Unit, Donka University Hospital, Faculty of Medicine, University of Conakry, Conakry, Guinea; ^2^Laboratory of Anatomo-Pathology/Biopathology, Centre Léon Bérard, Lyon, France; ^3^Laboratory of Anatomo-Pathology, University Hospital Centre of Donka, Faculty of Medicine, University of Conakry, Conakry, Guinea; ^4^Molecular Biology, Department of Basic Sciences, Faculty of Medicine, University of Conakry, Conakry, Guinea

## Abstract

A 30-year-old HIV positive woman presented with a multifocal mass tumour associated with axillary and lateral-cervical lymphadenopathy in the right breast. Laboratory examination of the biopsy confirmed a case of mammary Burkitt's lymphoma with a nodular infiltration of the breast. Antiretroviral treatment and chemotherapy were effective to control the tumour. Although Burkitt's lymphoma rarely involves the breasts, it should be considered during routine breast examination of African woman.

## 1. Introduction

Burkitt's lymphoma (BL) is one of the most common childhood cancers in Sub-Saharan Africa [1]. It occurs frequently in the regions where an endemic malaria infection is juxtaposed to chronic infection of the human gammaherpesviruses Epstein-Barr Virus (EBV). The disease is characterized by the fastest growth of B-cell non-Hodgkin lymphoma with monomorphic intermediate size cells.

According to WHO, BL can be classified into three clinical entities: the endemic, the sporadic, and the immunodeficiency-related BL [2]. The endemic BL which is frequent in Africa and is frequently associated with EBV infection occurs often as jaw or periorbital swelling. A proportion of abdominal presentation occurred also regardless of the presence of tumour at the jaw. The sporadic BL rarely associated with EBV has often abdomen localization; immunodeficiency-associated BL is frequent in HIV-infected patients. Large case-series have since the original observation confirmed the presence of EBV in endemic Burkitt's lymphoma cells.

Accordingly, virus has been demonstrated in more than 95% of investigated endemic Burkitt's lymphoma cases [3]. In contrast, the prevalence of EBV in sporadic Burkitt's lymphoma in general appears to be less than 30% [3, 4], albeit with considerable variation in reported estimates ranging between 15 and 88% [5]. In AIDS-related Burkitt's lymphoma, the prevalence of EBV is 25 to 50%. This proportion is near 100% in equatorial African BLs [4, 6].

According to molecular signature, BLs, regardless of EBV or HIV statute, are characterised by various alterations including mutations or chromosomal translocations affecting key cellular regulatory genes. Hence, based on genomic studies [7], there are BLs with c-MYC translocation (mBL), intermediate and non-c-MYC translocation. BLs with c-MYC translocation are characterized by the chromosomal rearrangements involving chromosomes 8 and 14, chromosomes 8 and 22, or chromosomes 2 and 8 which can lead to MYC activation and cellular growth transformation. Activation of c-MYC by overexpression or other means can lead to proliferation or apoptosis depending on the cellular microenvironment such as serum or growth-factor deprivation, nutrient privation, p53 status, or virus infection [8–10]. Activation of MYC can significantly contribute to the cell growth proliferation and transformation in the presence of alterations of other key regulatory genes such as P53. In BL, the conjunction of MYC activation, EBV infection, or other cellular gene alterations can drive B-cell to uncontrolled proliferation and tumour formation.

Primary breast lymphoma is a very rare disease accounting for 0.04–0.5% of all breast cancer cases, about 0.5% of all non-Hodgkin lymphomas [11]. It occurs often in pregnant, lactating women and HIV sero-positive patients [12, 13].

Here, we present a case of primary breast Burkitt's lymphoma (PBBL) in a HIV-infected Guinean young woman with the literature review.

## 2. Case Report

A 30-year-old Guinean woman who experienced a gradual increase of her right breast mass for 1 month consulted our unit on 17 July 2012. She was married to a polygamous man with a second wife. She had two pregnancies to term and had last been pregnant in her 26s. She experienced an allergy to quinine.

**Figure 1:**
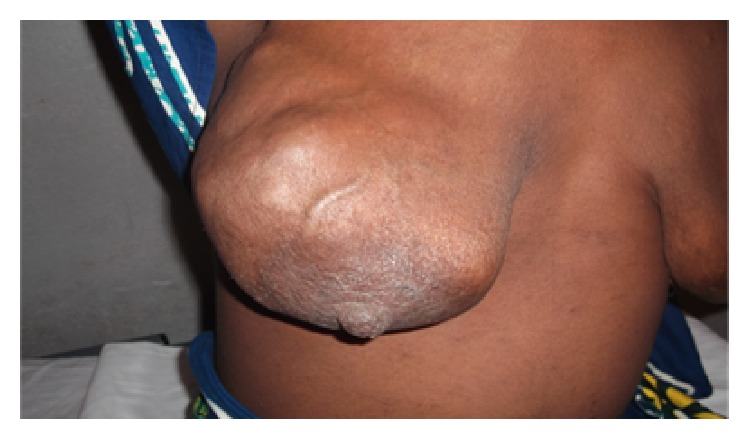
Multilobed mass of the right breast in a young woman of 40 years infected with HIV.

Physical examination indicated a large mass of 17 cm in the right breast. The tumour was multilobed, firm, and associated with mobile axillary and laterocervical lymphadenopathies with variable size no more than 3 cm (Figure 1). The patient was afebrile and had a hemoglobin level of 8.9 g/dL.

Ultrasound examination showed multiple hypoechogenic masses of varying sizes. Fine needle aspiration (FNA) suspected proliferation of lymphoma type of the breast. There was no deep lymph node involvement, and no mediastinal or abdominal location after chest X-rays and abdominal ultrasound examination, respectively. Serology was positive for the human immunodeficiency virus (HIV) type 1. CD4 count could not be determined due to the lack of reagent in the antiretroviral therapy centre. Bone marrow aspiration was not performed.

**Figure 2:**
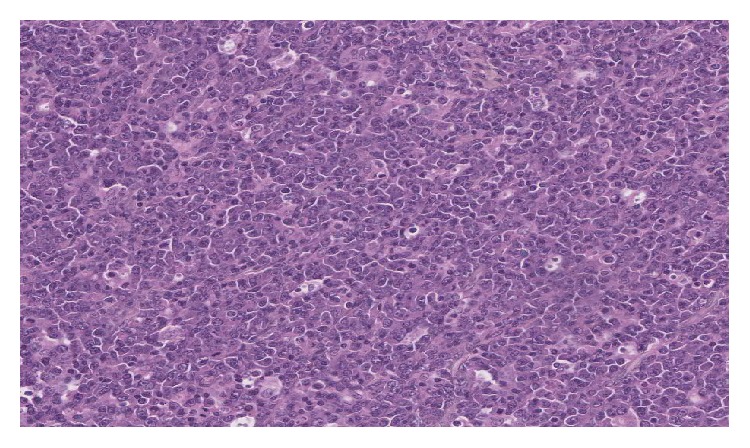
Breast lymphoma, microscopic aspects, HPS ×20.

**Figure 3:**
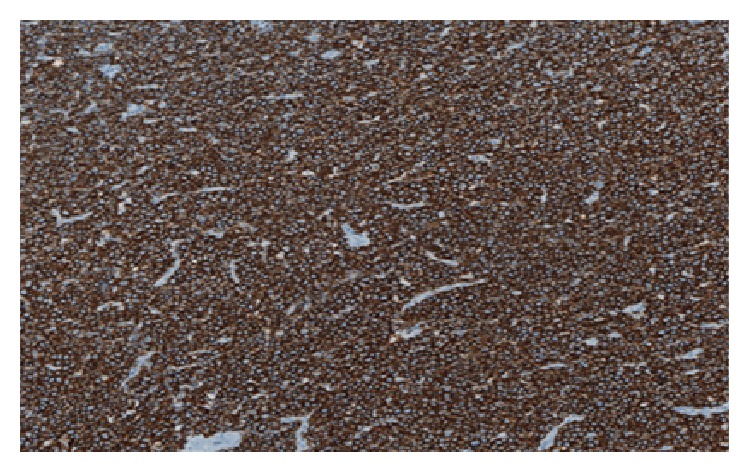
Breast lymphoma stained with anti-CD20 (×10).

**Figure 4:**
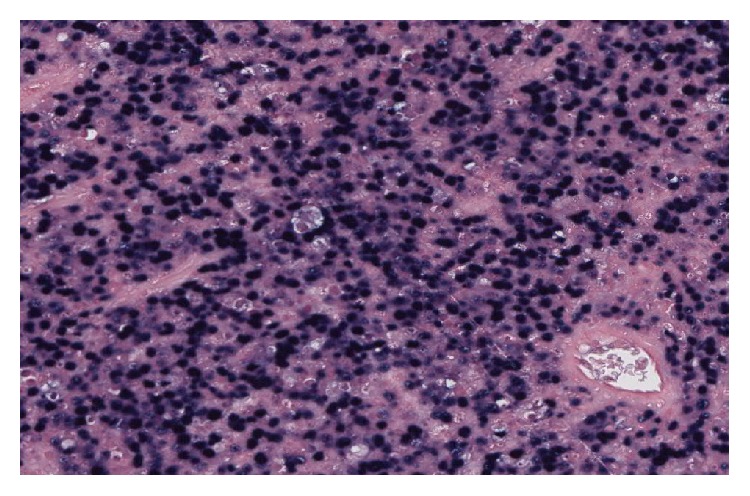
Epstein Barr Virus EBER staining in BBL.

**Figure 5:**
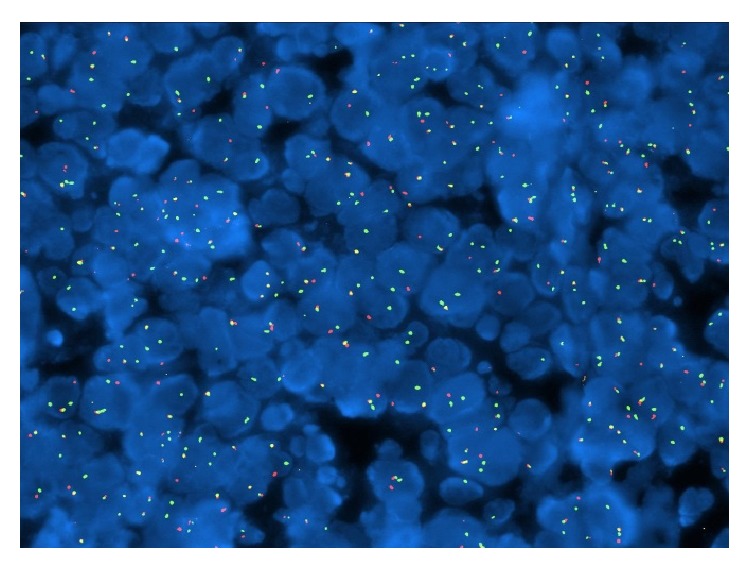
MYC Break Apart: complex and unusual rearrangement of MYC gene. Probe used: ZytoVision ZytoLight SPEC CMYC Dual Color Break Apart Probe.

Microscopic examination after biopsy had objectified tumour proliferation of blast cells containing a round nucleus with dense chromatin, sometimes with a nucleolus in the centre. Mitoses were numerous (43 mitoses per 10 fields at objective X40) accompanied by numerous apoptotic bodies. The field also contains small lymphocytes, polymorphonuclear neutrophils, and tangible macrophages bodies. The specimen did not reveal any residual nodal structure or breast parenchyma (Figure 2). Immunohistochemistry showed a clear staining of the tumour ample with the anti-CD20 antibody, indicating the B-cell origin of the cells (Figure 3). Few cells showed positive staining with anti-BCL2. It was difficult to interpret the staining using other antibodies probably due to the fixation process. Thus, heterogeneous labelling with edge effect was observed for anti-CD10, anti-BCL6, anti-MUM1, and anti-Ki67. The use of anti-EMA, anti-pan CK AE1 and AE3 did not identify the epithelial structures. The* in situ* hybridization performed later revealed clearly the presence of EBV in the tumour sample (Figure 4) and showed a complex and unusual MYC gene rearrangement (Figure 5).

It was concluded that the patient suffers from a PBBL localized in the right breast and characterized by a diffuse aggressive large B-cell CD20 + stage IIE associated with infection by Epstein-Barr virus (EBV) in the context of HIV infection.

The patient was then subjected in hospital to antiretroviral (ARV) treatment and chemotherapy. After one session of cure using COP protocol (cyclophosphamide 600 mg/m² day 1, vincristine 1,4 mg/m² day 1, and prednisolone 40 mg/m² days 1–5), a net regression of the tumour and associated lymph nodes was observed. After a second cure of chemotherapy, the patient was permitted to leave the hospital for ambulatory monitoring. Unfortunately, the patient did not show up regularly in the hospital for the follow-up and was lost of view with no reference. It was reported five months later that she died at home after developing back pain and paraplegia.

## 3. Discussion

PBBL is very rare disease and accounts for about 0.4 to 0.53% of all breast cancers [14]. In Sub-Saharan Africa where BL is the most common childhood neoplasia, the localization of this type of lymphoma in breast is not either a frequent event. Indeed, only three cases of PBBL have been reported in rare localizations of BL in Ivory Coast [15].

Between 2007 and 2012, the present case of primary breast BL was the only one identified among a series of 278 histologically with confirmed breast cancer collected in our surgical oncology unit. It represents 0.4% of all breast cancers recorded from 2007 to 2012 in our unit (Traore et al., Oral communication 3rd congress of SOGUIMES in Conakry). Overall, the case described in this study is the second reported case of PBBL in Guinea [16]. In both cases, the patient was young and female. However, the patient described in the current study was HIV positive. Thus, this is the first report of a case of HIV seropositive woman affected by PBBL in Guinea. In Guinea, the prevalence of HIV in general population is 1.5% [17]. There are no data on BL in adults in Guinea.

The clinical presentation of the mass in the breast is reminiscent of a phyllodes tumour [14]. However, the presence of axillary and lateral cervical lymphadenopathy excludes this possibility. We did not perform axillary or subclavicular nodes sampling, but these nodes have regressed completely with breast mass after chemotherapy. Bilateral forms are usually described at puberty or during breastfeeding [18].

Fine needle aspiration biopsy (FNAB) is a reasonably effective method of diagnosing BL and is a valuable diagnostic technique that allows material to be collected for the ancillary studies (immunophenotypic, molecular, and cytogenetic studies) [19]. Here, the diagnosis was confirmed by the morphological description of biopsy specimens, the detection of CD20 marker, and the presence of EBV. The presence of CD20, EBV and chromosomal alterations involving MYC are consistent with a diagnosis of African BL or African HIV + BL. However at this stage, one cannot exclude a case of a diffuse large B-cell lymphoma which can be difficult to separate morphologically from BL [7].

The prognosis of PBBL is characterized by the rapid clinical remission after chemotherapy [16, 20], provided that the patient completes the treatment. In addition, antiretroviral therapy may help to better tolerate the side effects of chemotherapy. Our patient was receiving ARV treatment, but she died from tumour progression because she had not continued chemotherapy. Thus, even rare in our clinical setting, the cases of PBBL should be considered during routine breast examination of African woman.
